# Risk factors for scabies in hospital: a systematic review

**DOI:** 10.1186/s12879-024-09167-6

**Published:** 2024-03-26

**Authors:** Dong-Hee Kim, Yujin Kim, Sook Young Yun, Hak Sun Yu, Hyun-Chang Ko, MinWoo Kim

**Affiliations:** 1 College of NursingᆞResearch Institute of Nursing Science, Pusan National University, Mulgeum-eup, Yangsan-si, Gyeongsangnam-do Korea; 2 College of Nursing, Pusan National University, Mulgeum-eup, Yangsan-si, Gyeongsangnam-do Korea; 3 Department of Parasitology and Tropical Medicine, School of Medicine, Pusan National University, Mulgeum-eup, Yangsan-si, Gyeongsangnam-do Korea; 4 Department of Dermatology, School of Medicine, Pusan National University, Mulgeum-eup, Yangsan-si, Gyeongsangnam-do Korea; 5 Department of Biomedical Convergence Engineering, Pusan National University, Mulgeum-eup, Yangsan-si, Gyeongsangnam-do Korea

**Keywords:** Scabies, Systematic review, Hospital, Risk factors

## Abstract

**Background:**

Annually, 175.4 million people are infected with scabies worldwide. Although parasitic infections are important nosocomial infections, they are unrecognized compared to bacterial, fungal, and viral infections. In particular, nonspecific cutaneous manifestations of scabies lead to delayed diagnosis and frequent nosocomial transmission. Hospital-based studies on the risk factors for scabies have yet to be systematically reviewed.

**Methods:**

The study followed the PRISMA guidelines and was prospectively registered in PROSPERO (CRD42023363278). Literature searches were conducted in three international (PubMed, Embase, and CINAHL) and four Korean (DBpia, KISS, RISS, and Science ON) databases. We included hospital-based studies with risk estimates calculated with 95% confidence intervals for risk factors for scabies infection. The quality of the studies was assessed using the Joanna Briggs Institute critical appraisal tools. Two authors independently performed the screening and assessed the quality of the studies.

**Results:**

A total of 12 studies were included. Personal characteristics were categorized into demographic, economic, residential, and behavioral factors. The identified risk factors were low economic status and unhygienic behavioral practices. Being a patient in a long-term care facility or institution was an important factor. Frequent patient contact and lack of personal protective equipment were identified as risk factors. For clinical characteristics, factors were categorized as personal health and hospital environment. People who had contact with itchy others were at higher risk of developing scabies. Patients with higher severity and those with a large number of catheters are also at increased risk for scabies infection.

**Conclusions:**

Factors contributing to scabies in hospitals range from personal to clinical. We emphasize the importance of performing a full skin examination when patients present with scabies symptoms and are transferred from settings such as nursing homes and assisted-living facilities, to reduce the transmission of scabies. In addition, patient education to prevent scabies and infection control systems for healthcare workers, such as wearing personal protective equipment, are needed.

**Supplementary Information:**

The online version contains supplementary material available at 10.1186/s12879-024-09167-6.

## Background

Scabies is an infectious disease caused by exposure to *Sarcoptes scabiei varietas hominis*. It spreads through close skin-to-skin contact with an infected person [1]. Infection with scabies causes symptoms, such as itching that worsens at night, rash, excoriated papules, eczematous lichenified plaques, and nodules [2, 3]. Secondary bacterial infections are complications that cause significant morbidity and mortality [4]. Scabies infection affects physical symptoms and quality of life, such as restriction of leisure activities and stigma [5, 6]. It occurs worldwide, and its prevalence was estimated at 175.4 million in 2017 [7]. The World Health Organization (WHO) classified scabies infection as a “neglected tropical disease” common in all races and classes [8], meaning that a deep understanding of the social burden caused by scabies infection is needed. Scabies is a major public health problem to be addressed [9].

Although parasitic infections play a significant role in nosocomial infections, awareness of parasitic infections is generally low compared to bacterial, fungal, and viral nosocomial infections. Consequently, diagnosis and treatment are significantly delayed [10]. The period from the onset of the first symptoms to diagnosis was more than 2 weeks in 46.1% of the patients [11]. Scabies transmission occurs when the diagnosis is delayed. The average number of infected patients per scabies outbreak was reported to be 18, and the average number of infected hospital care workers was 39, indicating high intrahospital transmission of scabies [12]. Therefore, prompt diagnosis and treatment are necessary.

However, nonspecific skin symptoms are more common in scabies than in typical features, such as burrows. As the clinical morphology of scabies is diverse [2, 13], it may be difficult to differentiate the diagnosis based on skin symptoms alone. Scabies is difficult to diagnose because it show a variety of clinical symptoms and severities [14], and unfamiliar atypical symptoms can lead to misdiagnosis by medical personnel [12]. Therefore, asking about risk factors for scabies, such as one’s living environment and skin symptoms, can provide insight into the diagnosis [15, 16]. In addition, early diagnosis questionnaires assessing risk factors play an important role in screening individuals for diseases [17, 18]. Hospital-based risk factors for scabies can also help distinguish scabies outbreaks.

To the best of our knowledge, systematic reviews of risk factors for scabies infection have primarily focused on community-based studies. Therefore, we aimed to identify the risk factors for patients with scabies in hospital settings, those who were diagnosed during outpatient visits, hospital admissions, or acquired within the hospital. Through this systematic review, we present the factors that help diagnosing scabies, with the intention of contributing to reducing disease transmission and burden.

## Methods

### Search strategy

The current systematic review was registered with the PROSPERO International Prospective Register of Systematic Reviews (ID: CRD42023363278). The study’s preliminary search was conducted on September 29, 2022, and the search was conducted from February 2 to 4, 2023. We searched the literature from the international PubMed, Embase, and CINAHL databases and the Korean domestic DBpia, KISS, RISS, and Science ON databases. The search terms were combinations of each database’s natural and control words for scabies, risk factors, and research settings (S1 Table). In addition, we added a search term to rule out animal research. The Boolean operator was used to combine terms and concepts. The search terms for the same concepts were combined with OR. The concepts were combined with AND or NOT. In addition to the academic databases, the study searched using Google Scholar by combining the words “scabies,” “risk factors,” and “hospital”.

### Article selection

The process of selecting studies is shown in S1 figure. We included a quantitative study of the risk factors for hospital infections of scabies. There were no restrictions on the year of publication or the age of the participants. The participants were not limited to patients but also included studies on hospital care workers.

First, duplicate papers were removed using the RefWorks program, and duplicate papers were excluded by manually checking them again. Next, two authors screened the titles and abstracts and excluded studies that did not address scabies. Next, we checked the full text of all retrieved studies to assess eligibility according to the inclusion and exclusion criteria. The inclusion criteria were studies (a) identifying risk factors for scabies infection; (b) hospital-based; (c) on the factors using calculated risk estimates with a 95% confidence interval; and (d) published either in English or Korean. The exclusion criteria were (a) Irrelevance (studies conducted in non-hospital settings, those not using statistical analysis for risk estimates with a 95% confidence interval, or those that do not identify risk factors); (b) conference abstracts, letters, posters, comments, or editorials; (c) protocols; (d) theses; (e) reviews; and (f) qualitative research.

In the case of Google Scholar, we combined search terms, such as scabies, risk factors, and hospitals. Then, we screened the titles and abstracts to extract our research topics and relevant studies and read the full texts to assess eligibility.

The process of selecting a study was conducted by two authors. If the two authors differed, the third author decided whether to include them.

### Search outcome

A total of 121 studies were found in the seven databases. Twenty duplicate studies were removed using RefWorks, and five duplicate studies were removed manually. In addition, two authors screened the titles and abstracts of 96 studies and excluded 25 studies that did not address scabies and one study involving animals. 70 studies were retrieved; therefore, we checked the full text and reviewed whether it was included based on the criteria of this systematic review. As a result, seven studies were included in this review.

In Google Scholar, we could not evaluate all the documents retrieved, so we reviewed 150 papers based on the searched list in order of accuracy. As a result, the full text of 17 papers dealing with risk factors for scabies was reviewed, and five studies were included. In conclusion, 12 studies were included in our systematic review using academic databases and Google Scholar.

### Data extraction

Data were extracted by two authors. Data extraction included a wide range of categories, such as author and publication information, study settings, study population, participant characteristics, diagnosis type, study design, prevalence, and risk factors evaluated in each study (S2 Table). Not clearly data were verified by emailing the authors.

### Quality assessment

Two authors independently performed a quality assessment using the Joanna Briggs Institute critical appraisal tools [19]. The designs of the studies in our review varied from cross-sectional, case-control, and cohort. We chose to use JBI’s critical appraisal tools because they offer quality assessment tools for all three designs. In summary, these tools assessed the quality of the participant selection, confounders, measures, and statistics. We judged the risk of bias to be low if 50% or more of the answers were “yes.” In contrast, the risk of bias was high if 50% or more of the responses were “no,” and the risk of bias assessment was if 50% or more of the “unclear” answers were judged as uncertain [19].

Table 1 shows the results of the qualitative assessment. Three studies did not meet the 50% “yes” criteria [20–22]. In addition, we emailed the original authors of one of the retrospective studies to confirm the concept of the study [23], such as cohort or case-control but did not receive a response. After discussion among the three authors, we determined that the purpose and design of the studies were acceptable for inclusion in our systematic review.

**Table 1:** Quality assessment

Cross-sectional studies																													
**1st author (year)**		Q1			Q2			Q3				Q4			Q5			Q6			Q7			Q8			Rate of Y		
Karaca Ural (2022) [27]		Y			Y			N				Y			N			N			Y			Y			62.5%		
Makigami (2009) [28]		Y			Y			N				Y			N			N			U			Y			50%		
Farhana (2018) [29]		N			Y			N				Y			N/A			N			Y			Y			50%		
Yeoh (2017) [30]		Y			Y			U				Y			N			N			U			Y			50%		
**Case-control studies**																													
**1st author (year)**		Q1		Q2			Q3			Q4			Q5		Q6		Q7			Q8		Q9			Q10			Rate of Y	
Lee (2021) [24]		Y		Y			Y			Y			Y		Y		Y			Y		Y			Y			100%	
Leistner (2017) [20]		N		Y			Y			N			Y		N		N			U		U			Y			40%	
Raza (2009) [25]		Y		Y			Y			N			Y		Y		Y			Y		Y			Y			90%	
Tsutsumi (2005) [31]		Y		Y			Y			Y			Y		N		N			Y		U			Y			70%	
Tufali (2021) [22]		N		N			N			N			Y		N		N			Y		U			Y			30%	
Wang (2012) [26]		Y		Y			Y			Y			Y		N		N			Y		U			Y			70%	
**Cohort study**																													
**1st author (year)**	Q1		Q2			Q3			Q4		Q5			Q6		Q7			Q8		Q9		Q10			Q11		Rate of Y	
Mulligan (2021) [21]	Y		Y			Y			N		N			U		Y			U		U		U			Y		45.5%	

Y: yes, N: no, U: unclear, N/A: not available

## Results

### Characteristics of the included studies

Table 2 presents the characteristics of the included studies. Twelve studies have been published on the factors related to the occurrence of scabies in hospitals. Five studies had case-control designs [20, 22, 24–26], four had cross-sectional designs [27–30], and three were retrospective analysis studies [21, 23, 31]. Looking at the hospital unit where the study was conducted, six studies were conducted on inpatients [21, 24, 26, 29–31], and two studies were conducted on outpatients [25, 27]. Four studies did not report the in-hospital settings in which they were performed [20, 22, 23, 28]. One study in an inpatient setting, and two studies that did not specify the study setting reported risk factors for nosocomial scabies infection [20, 28, 31]. Regarding the region where the study was conducted, Asia/Pacific had the most [22–31]. The number of participants ranged from 27 to 32,93,148. Prevalence was reported in studies designed with cross-sectional and retrospective studies. The prevalence of scabies ranged from 8.2 to 38.2%. One study reported scabies outbreaks by the hospital rather than by the number of patients, and 44.9% of the hospitals studied had scabies [28]. In one study, the risk factors for recurrence were investigated [23], five studies included both primary and recurrence [20–22, 29, 30], and six studies were not identifiable [24–28, 31]. The characteristics of individual studies included in the review can be found in the Supplementary information (S2 Table).

**Table 2:** Study characteristics

Study characteristics	N
**Design of study**	
Cross-sectional [27–30]	4
Case-control [20, 22, 24–26]	5
Retrospective [21, 23, 31]	3
**Region**	
Asia/Pacific [22–31]	10
Europe [20]	1
Americas [21]	1
**Study setting (the hospital setting to which the subjects belong)**	
Inpatient [21, 24, 26, 29, 30, 31^*^]	6
Outpatient [25, 27]	2
Not reported [20^*^, 22, 23, 28^*^]	4
**Number of subjects**	
0-100 [20, 31]	2
101–200 [24, 26, 30]	3
201–300 [23]	1
> 300 [21, 22, 25, 27–29]	6
**Diagnosis type of scabies**	
Recurrence [23]	1
All [20–22, 29, 30]	5
Unknown [24–28, 31]	6

^*^nosocomial infection

### Risk factors for scabies

Table 3 summarizes the results. In the outpatient setting, infrequent clothing changes [25] and rare bathing [25, 27] were identified as risk factors. In a study targeting inpatients, risk factors included residential type such as living in a nursing home [26], history of admission to long-term care facilities [24], and homelessness [21]. Among inpatients, walking conditions [31] and severity [26] were also revealed as risk factors. Among nosocomial scabies, contact [20], walking conditions [31], and hospital environment [28] were identified as risk factors. Four studies did not clearly report the hospital settings in which the study was conducted. Therefore, the overall integration of risk factors for all 12 studies was described by categorizing them into two groups: (a) personal and (b) clinical.

**Table 3:** Risk factors for scabies

Characteristics			Risk factors (Reference number)	Study Setting		
				**Outpatient**	**Inpatient**	**Not-reported**
Personal	**Demographic**	**Gender**	**Male** [23, 27]	**v** [27]		**v** [23]
		**Age**	**19–39, 40–64, above 65** [21]		**v**	
			**First-school children** [29]		**v**	
			**21–50** [22]			**v**
			**Age** increased [23]			**v**
		**Race**	**Asian&Pacific Islander, Native American** [21]		**v**	
		**Education**	**Low** [25]	**v**		
		**Season**	**Season** Winter [29]		**v**	
			**May to August** [23]			**v**
	**Economics**	**Economic status**	**Low** [29]		**v**	
		**Income**	**Average** [22]			**v**
		**Insurance status**	**No charge, uninsured, Medicaid** [21]		**v**	
		**Job/working status**	**Nonworking** [27]	**v**		
			**Student** [22]			**v**
			**Leave/temporary Duty** [25]	**v**		
	**Resident**	**Type**	**Rural** [27]	**v**		
			**Urban** [29]		**v**	
			**Living in a nursing home** [26]		**v**	
			**Prior long-term care facility admission** [24]		**v**	
			**Homeless** [21]		**v**	
			**Unit barracks** [25]	**v**		
		**Environment**	**Households** > 5 [30]		**v**	
			**Living condition** Poor [22]			**v**
		**Region**	**Western region of Saudi Arabia** [23]			**v**
	**Behavioral**	**Personal hygiene**	**Changing clothes < 2** times/week [25]	**v**		
			**Bathing < 1** time/day [25]	**v**		
			**No. of baths per month 9<** [27]	**v**		
			**Poor** [22]			**v**
		**Sharing**	**Beds** [25]	**v**		
			**Household accessories** [29]		**v**	
		**Contact**	**Disposable gloves are rarely used when examining patients** [20]			**v** ^*^
			**Holding the patient often** [20]			**v** ^*^
Clinical	**Individual clinical**	**Walking conditions**	**Ease of movement, without assistance** [31]		**v** ^*^	
			**Range of movement, out of the room but within the ward** [31]		**v** ^*^	
			**Bedridden status** [26]		**v**	
		**Severity**	**Acute Physiology and Chronic Health Evaluation(APACHE) II score, mean/range** [26]		**v**	
			**APACHE II score** ≧ 20 [26]		**v**	
			**Catheters** [26]		**v**	
			**Days of hospitalization, mean/range** [26]		**v**	
			**Expiration during study period** [26]		**v**	
		**Itching sign**	**Itching in close contact** [29]		**v**	
			**Itching in family/ colleagues** [25]	**v**		
	**Hospital environment**	**Units**	**Acute-care wards** [28]			**v** ^*^
			**Long-term care wards** [28]			**v** ^*^
		**Size of hospital**	**Size of the hospital** [28]			**v** ^*^
		**Infection** **control policy**	**Have regular preventive measures for scabies** [28]			**v** ^*^
			**Dermatological examination on admission** [28]			**v** ^*^
			**Treat all suspected patients with scabicides** [28]			**v** ^*^

^*^nosocomial infection

### Personal factors

Among the risk factors reported to increase the occurrence of scabies, those closely related to an individual’s life were classified as personal characteristics. These include demographic, economic, resident, and behavioral characteristics.

### Demographic characteristics

Demographic factors were reported for sex, age, race, and education level. Two studies reported that men were more at risk for scabies infection [23, 27]. The age reported as a risk factor in each study was different. In the study by Farhana et al. (2018), first-school children were at risk for scabies infection [29], whereas Tufali (2021) reported that the risk factor was individuals aged 21–50 years [22]. Mulligan et al. (2021) found that scabies was more prevalent in the 19–39, 40–64, and 65 + age groups than in the 18 and under age groups [21], and Ahmed et al. (2019) found that the risk of infection increased as age increased [23]. In one study that identified race as a risk factor, Asians, Pacific Islanders, and Native Americans were at higher risk of scabies [21]. In one study, education level was reported; Raza et al. (2009) found that a lower education level increased the incidence of scabies [25].

### Economics characteristics

Five studies reported factors belonging to the economic category. In most studies, low economic status was a risk factor for scabies infection. For example, Farhana et al. (2018) found that a low economic level was a risk factor for scabies [29], while Tufali (2021) reported that the risk of scabies infection was greater in the case of the average income level [22]. In this study on the occurrence of scabies according to insurance status, patients with no charge, uninsured, and Medicaid insurance had a high risk of scabies infection [21]. Three studies reported an association between job and working status and scabies; Karaca Ural et al. (2022) was nonworking [27], and Tufali (2021) showed that scabies frequently occurred in students [22]. Raza et al. (2009) reported that soldiers on leave or temporary duty were vulnerable to scabies [25].

### Resident characteristics

Six studies reported that the risk of scabies infection increased depending on the residence type. One study found that living in a rural area was a risk factor [27], and another study reported that living in an urban area was a risk factor [29]. Living in a nursing home or a long-term care facility, and admission were also found to be risk factors for scabies [24, 26]. One study showed that scabies occur more frequently in people experiencing homelessness [21]. In a study conducted in a military hospital, living in unit barracks increased the risk of scabies infection [25]. In terms of residence environment, it has been found that the incidence of scabies rises when there are more than five members in the family [30]. Another study reported that the incidence of scabies was higher when the living condition was poor than when it was good [22]. In a study conducted in Saudi Arabia, there was a difference in the risk of occurrence depending on the place of residence [23].

### Behavioral characteristics

Factors related to behavioral characteristics were reported in five studies. Two studies on personal hygiene reported that fewer bathing times increased the risk of scabies infection [25, 27]. In addition, infrequent changes in clothes or poor personal hygiene were associated with an increased risk of scabies infection [22, 25]. Sharing was a risk factor for scabies infection, including beds [25] or household accessories such as beds, towels, and clothes [29]. In one study of hospital care workers, the relationship between care practice, and the occurrence of scabies was investigated. Scabies occurred more frequently when holding the patient and failing to wear gloves during physical examinations [20].

### Clinical factors

Clinical characteristics were described separately from personal characteristics among the risk factors for scabies. Clinical characteristics related to those of the human participants and the environmental characteristics of the hospital.

### Individual clinical characteristics

Four studies reported the relationship between the individual clinical characteristics of the study participants and scabies infection. Two contradictory results were reported for gait status or an individual’s ambulation. One study reported being able to move without assistance or range of movement out of the room but within the ward as a risk factor [31]. However, studies have shown an increased risk of scabies infection in bedridden patients [26]. One study addressed factors related to patient severity. A high average APACHE II score > 20, catheters, mean days of hospitalization, and expiration during the study period were risk factors for scabies [26]. In addition, the risk of scabies infection was greater when the patient was in close contact with family or colleagues experiencing itching [25, 29].

### Hospital environment characteristics

Only one study reported an association between the hospital setting or environment and the incidence of scabies. For example, the risk of infection was greater in acute care and long-term care wards. Another factor was hospital size or infection control policies, such as “dermatological examination on admission” and “regular preventive measures for scabies,” have been identified as risk factors for scabies infection [28].

## Discussion

We conducted a systematic review to identify the risk factors for scabies to prevent infection; in this study, 12 hospital-based studies identified risk factors for scabies. The studies were divided into personal and clinical risk factors and confirmed that scabies was a multifactorial occurrence.

Personal factors were grouped into demographic, economic, residential, and behavioral risk factors. Among the demographic characteristics, men were identified as at a greater risk for scabies than women. Community-based studies that support this result were conducted in Ethiopia [32] and Liberia [33], and a study in Nigeria contradicted these results [34]. However, care must be taken in interpreting gender, as it may be a result of the cultural characteristics of the surveyed region or a result that does not reflect the population. In the case of education level, when the education level was low, the understanding of preventive behavior and treatment for infectious diseases was low, and the risk of scabies was greater. However, when the education level was higher and income was higher, the risk of scabies outbreaks was lower because patients could have more frequent medical consultations [32]. Demographic characteristics can be recognized in screening for the diagnosis and identification of patients at risk for scabies infection.

Each of the five studies had different standards for economic levels, such as income, economic level, type of insurance, and occupational status. Taken together, it was judged that the risk of scabies is greater for individuals with lower economic income. A study identifying the risk factors for scabies in the community also reported that the lower the annual income level was, the greater the incidence of scabies [32, 34, 35]. People with low economic status often have poor living conditions, which can lead to the transmission of scabies [35]. In addition, treatment-related costs affect family income, and daily hospital visits are costly; economic problems are both a cause and consequence of illness [36]. These studies suggest that economic level should be considered a major factor in the early screening for scabies.

Regarding the type of residence, each study reported various results making it difficult to merge; however, nursing homes and long-term care facilities show similar residence characteristics; for example, patients live in unavoidable contact for a long time. Closed communities experience higher incidences of scabies [37, 38]. Moreover, scabies in nursing homes are particularly asymptomatic or atypical, reaching 51% [37]. These factors may increase silent transmission, suggesting that hospital admissions or visits may not be aware of scabies infection. Therefore, patients who visit hospitals and have a history of living in a nursing home or long-term care facility should be thoroughly screened for the occurrence of scabies.

Among the behavioral factors, unsanitary behavior and sharing or contacting objects with others were identified as risk factors. While Dagne et al.’s (2019) and Yassin et al.’s (2017) studies in the community showed the same results [39, 40], the results of the meta-analysis reported by Azene et al. (2020) were not significant [41]. Hygiene behavior due to a lack of facilities in the home is difficult to modify, but it is a factor that can be sufficiently changed in terms of education. Previous studies have reported that the incidence of scabies decreased after education on personal hygiene [42, 43]. Therefore, educational programs and preventive activities are needed to prevent the occurrence of scabies. Sharing or touching objects with others was also found to be a risk factor for scabies. Healthcare providers were responsible not only for treating scabies but also for preventing their spread. This is based on limiting contact with ticks, early diagnosis, and appropriate health education [44]. When a patient with scabies symptoms visits a hospital, it is important to screen quickly. However, if immediate diagnosis or screening among patients with risk factors is not possible, it is necessary to educate patients to use individual objects during the incubation period of at least two weeks. Moreover, frequent contact with patients or failure to wear protective equipment were identified as risk factors for medical staff and hospital personnel. An infection control system, such as wearing personal protective equipment for infection protection by medical personnel dealing with symptomatic patients, should be established.

Clinically related risk factors were divided into individual medical characteristics and hospital environments. Among the individual clinical characteristics, there were two studies on activity status, and the results of each study were contradictory. In a study by Tsutsumi et al. (2005), the incidence of infection was higher when there was frequent movement [31]. As scabies infection occurs through contact, Hay et al. (2012) showed the same outcome [38]. In contrast, Wang’s (2012) study indicated that patients in a bedridden state were at greater risk of occurrence [26]. Patients in a bedridden state may be vulnerable to infections owing to reduced immunity [45, 46]. Immunosuppression, such as immunodeficiency, the presence of immunosuppressive factors, or malnutrition, increases the incidence of scabies [47–49]. In addition, nosocomial scabies was associated with an immunocompromised status [12]. Moreover, bedridden patients may have difficulty expressing pruritus, a major symptom of scabies. Skin-to-skin contact is the most common transmission route for scabies, and even healthy individuals can become infected with scabies through contact [1, 38]. In this context, the possibility of healthcare workers transmitting infection to bedridden patients can also be considered. Therefore, regular skin screening in hospitals for bedridden patients is necessary. Wang et al. (2012) reported that patients with higher APACHE scores, more catheters, and longer hospital stays were more susceptible to scabies infection. This suggests a correlation between the severity of the patient’s condition and scabies infection [26]. Only one study identified patient severity as a risk factor for scabies, so further research is needed.

In two studies, itching in close family members or acquaintances was significantly associated with scabies [25, 29]. This is supported by findings from previous community studies [34, 41]. Therefore, when taking the history of patients with skin diseases, checking whether a family member has pruritus is an important question in diagnosing scabies.

Hospital environmental risk factors were found in only one study, making integration difficult. As environmental factors also affect the occurrence of scabies [38], follow-up studies on the degree of scabies occurrence according to hospital characteristics are necessary. Additionally, regarding environmental risk factors, patients in long-term wards were more vulnerable to scabies [28]. Therefore, we again emphasize screening patients hospitalized for a long time.

Recently, active research has been conducted on the early screening of patients with risk factors. For example, Quéreux et al. (2010) developed a self-assessment questionnaire using risk factors for melanoma [50]. Nichol et al. (2019) created a self-screening tool using risk factors for hand dermatitis among healthcare workers [51]. These screening tools reduce the efforts of medical staff by helping to select subjects for full skin examination and play a significant role in determining individuals with the possibility of disease detection [17, 18]. Diagnostic programs incorporating new technologies, such as a deep-learning system that predicts diseases using images of skin lesions, are also being developed [52, 53]. Even in deep learning, genetic, life, and demographic risk factors can be input and combined as variables to achieve better model performance. In addition, risk factors can be input as data variables [53, 54]. The risk factors for scabies identified in our study can be used to develop various screening tools or programs.

Of the studies included in our systematic review, 10 of the 12 were conducted in Asia/Pacific [22–31]. Most research on scabies has focused on countries with a low-to-moderate United Nations Human Development Index (HDI) or disadvantaged populations in countries with high HDI. Thus, nonindigenous population data for North America, most of Europe, and Australia were lacking [55]. However, the prevalence and incidence of scabies have increased in high-sociodemographic index countries and high-income North America [7]. Therefore, further research is needed to identify the risk factors for scabies outbreaks in countries, such as North America and Europe, which have been less studied.

We attempted to integrate the results based on the hospital settings in which the study was conducted. For outpatients, personal characteristics, such as lack of hygiene, emerged as risk factors [25, 27]. In contrast, inpatients living in communal settings [21, 24, 26] were vulnerable to scabies infection, and clinical factors such as severity [26] and walking conditions [31] tended to be more pronounced compared with outpatients. These results provide information on the factors that should be emphasized in screening for scabies tailored to each outpatient and inpatient setting. In nosocomial scabies, characteristics classified as clinical factors, such as contact [20] and hospital environment [28], were highlighted as risk factors. Based on these results, to prevent the spread of scabies within hospitals, wearing protective equipment is necessary to prevent direct contact with patients and establish infection control policies within hospitals. However, only one study reported factors such as severity, walking conditions, and hospital environment. This indicates a lack of studies on the risk factors associated with scabies occurrence and suggests that more research should be conducted.

## Conclusion

We conducted a systematic review that included 12 studies to identify risk factors for hospital-based scabies. Factors contributing to scabies in hospitals range from personal to clinical. The results of this systematic review provide a basis for the proactive identification of individuals vulnerable to scabies outbreaks and for preventive measures to be taken. Based on these results, we suggest that patients presenting with symptoms of scabies in settings, such as long-term care facilities or nursing homes, should be screened for scabies to reduce transmission. In addition, patient education to prevent scabies and infection control systems for healthcare workers, such as wearing personal protective equipment, is needed.

### Electronic supplementary material

Below is the link to the electronic supplementary material.

**Supplementary Material 1: Table S1.** Search strategies according to each database

**Supplementary Material 2: Table S2.** Characteristics of the included studies

**Supplementary Material 3: Table S3.** PRISMA Checklist

**Supplementary Material 4: Fig S1.** PRISMA flow diagram

## Data Availability

All relevant data are within the manuscript and its Supporting Information files.
