# Supplementary material for: Risk factors for scabies in hospital: a systematic review
Source: BMC Infect Dis. 2024 Mar 26;24:353. doi: 10.1186/s12879-024-09167-6 (PMC10993523; doi:10.1186/s12879-024-09167-6)
Supplement: Supplementary file 1 — Supplementary Material 1: Table S1. Search strategies according to each database [file 12879_2024_9167_MOESM1_ESM.docx]

**S1 Table. Search strategies according to each database**

| Database | Search | Concept | Search terms | No. of ref |
| --- | --- | --- | --- | --- |
| Pubmed | #1 | Sacbies | scabies[MH] OR Sarcoptes scabiei[MH] OR scabie*[TIAB] OR Sarcoptes scabie*[TIAB] | 5,402 |
|  | #2 | Risk factors | risk factors[MH] OR risk factor*[TIAB] OR determinant factor*[TIAB] OR prevention factor*[TIAB] OR associated factor*[TIAB] OR predictive factor*[TIAB] OR vulnerability factor*[TIAB] OR sociodemographic factor*[TIAB] OR socioeconomic factor*[TIAB] | 1,389,099 |
|  | #3 | Settings | Hospitals [MeSH Terms] OR hospitalization[MeSH Terms] OR inpatients[MeSH Terms] OR outpatients[MeSH Terms] OR health Facilities[MeSH Terms] OR hospital*[TIAB] OR health facilit*[TIAB] OR inpatient*[TIAB] OR outpatient*[TIAB] OR care center*[TIAB] | 2,303,821 |
|  | #4 | Condition | (animals) NOT (animals AND humans) | 4,717,458 |
|  | #5 | Search | ((#1 AND #2) AND #3) NOT #4 | 70 |
| EM  -base | #1 | Sacbies | 'scabies'/exp OR 'Sarcoptes scabiei' OR 'scabie*' OR 'sarcoptes scabie*' | 8,355 |
|  | #2 | Risk factors | 'risk factors'/exp OR 'risk factor*' OR 'determinant factor*' OR 'prevention factor*' OR 'associated factor*' OR 'predictive factor*' OR 'vulnerability factor*' OR 'sociodemographic factor*' OR 'socioeconomic factor*’ | 1,739,170 |
|  | #3 | Settings | ‘Hospital’/exp OR ‘health care facility’/exp OR ‘Hospitalization’/exp OR ‘inpatient’/exp OR ‘outpatient’/exp OR hospital* OR　health facilit*　OR inpatient* OR outpatient* OR care center* | 1,442,386 |
|  | #4 | Condition | animals NOT (animals AND humans) | 870,455 |
|  | #5 | Search | ((#1 AND #2) AND #3) NOT #4 | 15 |
| CINAHL | S1 | Sacbies | scabies OR "Sarcoptes scabiei" OR scabie* OR "Sarcoptes scabie*" | 1,052 |
|  | S2 | Risk factors | "risk factor*" OR "determinant factor*" OR "prevention factor*" OR "associated factor*" OR "predictive factor*" OR "vulnerability factor*" OR "sociodemographic factor*" OR "socioeconomic factor*" | 642,059 |
|  | S3 | Settings | hospitals OR hospitalization OR inpatient OR outpatients OR health Facilities OR hospital*OR health facilit* OR inpatient*OR outpatient* OR care center* | 938,835 |
|  | S4 | Condition | animals NOT (animals AND humans) | 191,953 |
|  | S5 | Search | (S1 AND S2 AND S3) NOT S4 | 20 |

No: number, ref: references
