# Supplementary material for: Risk factors for scabies in hospital: a systematic review
Source: BMC Infect Dis. 2024 Mar 26;24:353. doi: 10.1186/s12879-024-09167-6 (PMC10993523; doi:10.1186/s12879-024-09167-6)
Supplement: Supplementary file 2 — Supplementary Material 2: Table S2. Characteristics of the included studies [file 12879_2024_9167_MOESM2_ESM.docx]

**S2 Table. Characteristics of the included studies**

| **1^st^ author (year)**  **/ Study location** | **Study setting** | **Study Population** | **No. of participants**  **(male %)**  **Mean ages(y) ± SD** | **Diagnosis type** | **Study design** | **Results**  Prevalence: n(%)  R: risk factors associated with scabies  (aOR or OR or cOR or aHR, 95%CI/ ref. group^*^) |
| --- | --- | --- | --- | --- | --- | --- |
| Ahmed (2019)/  Saudi Arabia | Information of patients registered  BESTCare database | Children age of  less than 14 years who were diagnosed with one or multiple episodes of scabies during the study period | 264(54.9)  6.7±3.95 | Recurrence | Retrospective study | R: **Age** increased (aHR, 1.036; 95% CI, 1.002–1.072)  **Sex** males (aHR, 1.734, 1.329– 2.262)  **Region** Western region of Saudi Arabia (aHR, 1.548, 1.115–2.151  **Season** May to August (aHR, 2.368, 1.706–3.288) |
| Farhana (2018)  / Pakistan | Dermatology department of the military hospital (inpatient) | All admitted patients who visited study hospital during the data collection period | 2,364(47.1)  Not reported | Primary & recurrence | Cross-sectional | Prevalence: 902 (38.2)  R: **Age FS** (1.51, 1.19-1.93)  **Residence** Urban (2.31, 1.95-2.76/ rural)  **Itching in close contact** Yes (683.86, 915.53-3097.01/ no)  **Sharing household accessories** Yes (36.53, 26.71-49.96/ no)  **Season** Winter (5.61, 4.67-6.74)  **Economic status^***^** Low 3.68 (3.09-4.39) |
| Karaca Ural (2022)  / Turkey | Dermatology  Clinic in hospital  (outpatients) | Patients applying to dermatology outpatient clinic between 1st and 30th April 2021 | 376(41.0)  Not reported | Unknown | Cross-sectional | Prevalence: 41(10.9)  R: **Residence** Rural (2.728, 1.325-5.557/ urban)  **Sex** Male (2.714, 1.365-5.451/female)  **Employment status** Nonworking (2.707, 1.256-5.833/ working)  **No. of baths per month** 9< (2.354, 1.057-5.243/ 9 ≥) |
| Lee  (2021)  / South Korea | Acute care teaching hospital  (inpatients) | The patients who have been admitted to a study hospital and have been diagnosed with scabies | 102(50.0 of case)  (case:control= 1:2)  61 ± not reported in case | Unknown | Case-control  (matched ages and sex, random sampling in control group) | R: **Prior Long-term care facility admission** (5.441, 1.460-20.272) |
| Leistner (2017)  / Germany | Acute care hospital  (nosocomial) | HCWs who have had contact with a patient with scabies | 27(70.4)  (case:control= 13:14)  45 in controls (median, IQR)  48 in cases (median, IQR) | Primary & recurrence | Case-control | R: **Disposable gloves are rarely used when examining patients** (9.789, 1.163-82.41)  **Holding the patient often** (8.151, 1.121-59.270) |
| Makigami  (2009)  / Japan | Psychiatric and long-term care hospitals  (nosocomial) | Either or both members of the Japanese Association of Psychiatric Hospitals or the Japanese Association of Long-term Care Hospitals.  The reported targets of nosocomial infections include inpatients, hospital personnel, or patients' family members. | 741^**^(Not applicable)  Not applicable | Unknown | Cross-sectional | Prevalence: 333 (44.9) ^**^  R: **Acute-care wards** (2.40, 1.52-3.79)  **Long-term care wards** (1.91, 1.34-2.72)  **Size of hospital** (1.30, 1.12-1.52)  **Have regular preventive measures for scabies** (4.06, 2.63-6.28)  **Dermatological examination on admission** (1.57, 1.09-2.28)  **Treat all suspected patients with scabicides** (1.95, 1.40-2.72) |
| Mulligan  (2021)  / US | Information of patients registered NIS from 2012 to 2016  (inpatients) | Patients diagnosed with scabies during hospital admission | 32,931,148(not reported)  (9,600 with scabies)  Not reported | Primary & recurrence | Retrospective analysis | Prevalence: 29.3 of every 100,000 inpatient admissions  R**: Ages** 19-39 (1.16, 1.06-1.27/ ≤18)  40-64 (2.05, 1.87-2.24/ ≤18)  ≥65 (1.36, 1.22-1.51/ ≤18)  **Race** Asian& Pacific Islander (1.13, 1.06-1.21/ white)  Native American (1.49, 1.21-1.83/ white)  **Housing status** Homeless (11.55, 10.65-12.53/ nonhomeless)  **Insurance status** No charge (1.39, 1.10-1.75/ medicare)  Uninsured (1.20, 1.10-1.31 / medicare)  Medicaid (1.36, 1.28-1.44) |
| Raza  (2009)  / Pakistan | Military hospital  (outpatients) | Male soldier showing symptoms of scabies or dermatology, visiting 3 dermatology outpatient departments | 400(100.0)  (case:control= 1:1)  29.17±7.13 in cases  29.19±7.13 in controls | Unknown | Case-control | R: **Itching in family/ colleagues (**52.89, 7.20-388.58)  **Bathing < 1 time/day** (4.07, 2.39-6.93)  **Changing clothes** < 2 times/week (3.67, 1.85-7.27)  **Low education** (3.22, 1.95-5.32)  **Residence in unit barracks** (3.08, 1.75-5.39)  **Leave/temporary duty** (3.02, 2.12-4.30)  **Sharing beds** (1.91, 1.26-2.92) |
| Tsutsumi  (2005)  / Japan | Dementia ward (nosocomial  inpatients) | All inpatients in the dementia ward during the outbreak period | 65(12.3)  81.6 ± 7.9 with scabies | Unknown | Retrospective epidemiologic study | Prevalence: 20 (30.8)  R: **Ease of movement, without assistance** (11.3, 2.9-44.8)  **Range of movement, out with the room but within the ward** (4.1, 1.4-12.5) |
| Tufail  (2021)  / Pakistan | Hospital | Patients who  visited in study hospital | 531(45.9)  (case:control= 1:2)  Not reported | Primary & recurrence | Case-control (unmatched) | R: **Age** 21-50 (1.70, 1.18-2.45)  **Occupation** Student (1.71, 1.12-2.62)  **Living condition** Poor (7.44, 3.29-16.82)  **Personal Hygiene** Poor (48.1, 11.45-202.1)  **Income** Average (1.72, 1.190-2.494) |
| Wang  (2012)  / Taiwan | Tertiary care, teaching hospital (inpatients) | Adults patients with a diagnosis of scabies after admission from June 2007 to June 2010 | 157(45.9)  (case:control= 1:2)  79.1 ± not reported in the case  71.82 ± not reported in controls | Unknown | Case-control  (matched ages and sex) | R: **Bedridden status** (6.72, 3.04-14.88)  **APACHE II score, mean/range** (1.25, 1.12-1.36)  **APACHE II score** ≧20 (11.88, 4.63-30.44)  **Cathethers**^#^ (9.05, 3.33-24.57)  **Living in a nursing home** (9.89, 4.41-22.18)  **Expiration during the study period** (7.98, 3.08-20.68)  **Days of hospitalization(mean/range)** ^##^ (1.05, 1.02-1.07) |
| Yeoh^###^  (2017)  / Australia | Pediatric ward  (inpatients) | Children and adolescents under the age of 16 were admitted to two hospitals | 158(54.4)  Not reported  (Median 3.6, IQR 0.9-7.4) | Primary & recurrence | Cross-sectional | Prevalence: 13 (8.2)  R: **Household** >5 (4.0, 1.1-14.0/ <5) |

No: nurmer; y: years, aOR: adjusted odds ratio, OR: odds ratio, aHR: adjusted hazard ratio, CI: confidence interval, ref.: reference, BESTCare: a large multi-center electronic health information system implemented in MNGHA(Ministry of National Guard Health Affairs), IQR: Interquartile Range, NIS: National Inpatient Sample, FS: first school children

^*^: Only listed if reference groups are reported

^**^: Number of hospitals participating in the survey

^***^: low (<15,000 Pak rupees/month), middle (15,000-50,000 Pak rupees/ month) and high (>50,000 Pak rupees/month)

^#:^ Catheters include nasogastric tube, Foley catheter, Port-A, and Hickman catheter

^##:^ All other results in this study (Wang, 2012) are univariable-regression reported, and only ‘Days of hospitalization’ is multivariable logistic regression reported.

^###^: reported only prospective case result
