# Supplementary figures and images for: Risk factors for scabies in hospital: a systematic review
Source: BMC Infect Dis. 2024 Mar 26;24:353. doi: 10.1186/s12879-024-09167-6 (PMC10993523; doi:10.1186/s12879-024-09167-6)

**S1 Figure. PRISMA flow diagram**

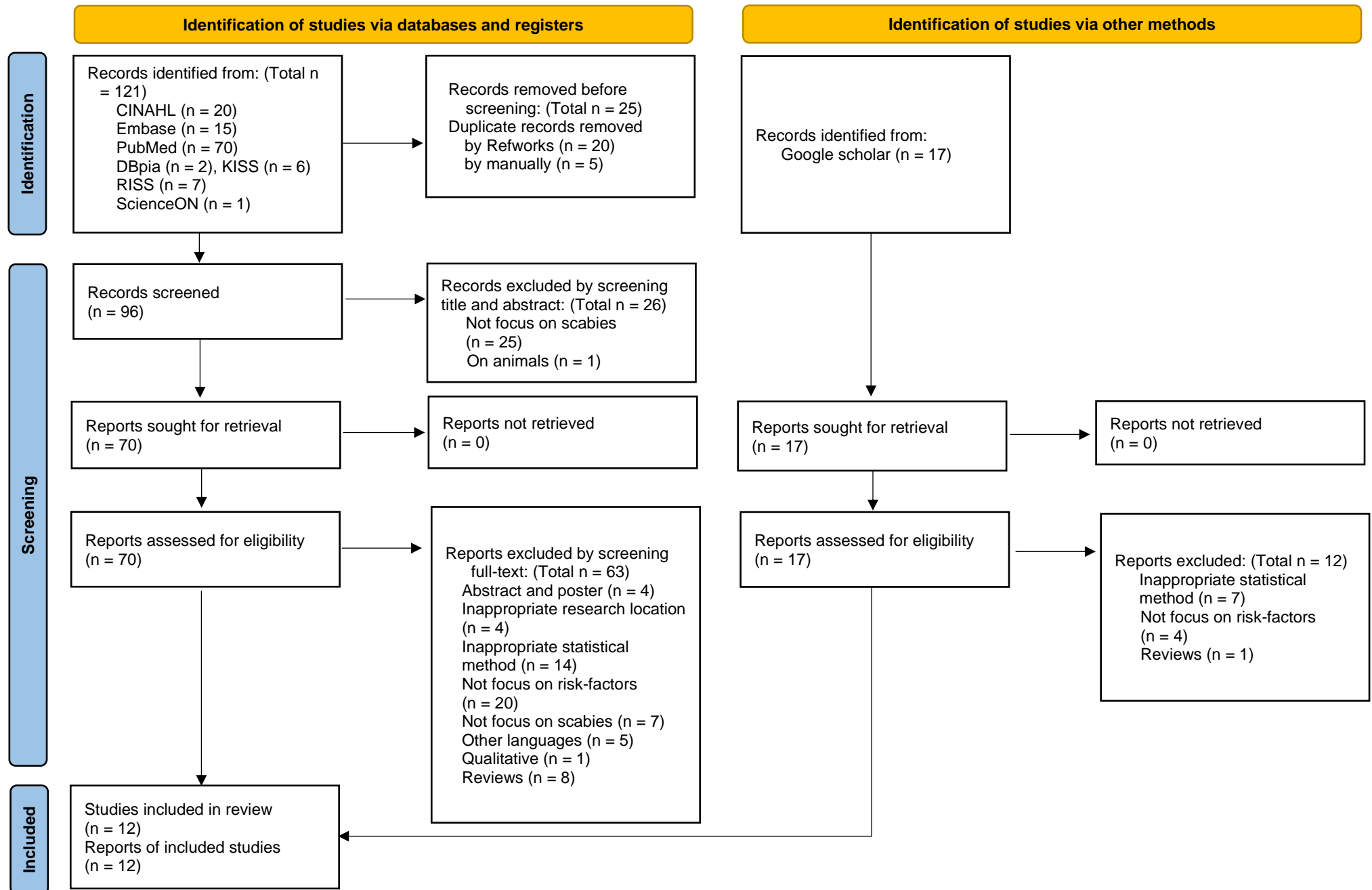

Supplement: Supplementary file 4 — Supplementary Material 4: Fig S1. PRISMA flow diagram [file 12879_2024_9167_MOESM4_ESM.pdf]
